# Supplementary material for: USP16 counteracts mono-ubiquitination of RPS27a and promotes maturation of the 40S ribosomal subunit
Source: eLife. 2020 Mar 4;9:e54435. doi: 10.7554/eLife.54435 (PMC7065907; doi:10.7554/eLife.54435)
Supplement: Supplementary file 1. [file elife-54435-supp1.docx]

| **Key Resources Table** | | | | |
| --- | --- | --- | --- | --- |
| **Reagent type (species) or resource** | **Designation** | **Source or reference** | **Identifiers** | **Additional information** |
| cell line (*Homo sapiens)* | HEK293 FlpIn T-REx HASt-GFP | (Wyler et al., 2011) DOI: [10.1261/rna.2325911](https://doi.org/10.1261/rna.2325911) |  |  |
| cell line (*Homo sapiens*) | HEK293 FlpIn T-REx ENP1-StHA | (Wyler et al., 2011) DOI: [10.1261/rna.2325911](https://doi.org/10.1261/rna.2325911) |  |  |
| cell line (*Homo sapiens*) | HEK293 FlpIn T-REx HASt-DIM2 | (Wyler et al., 2011) DOI: [10.1261/rna.2325911](https://doi.org/10.1261/rna.2325911) |  |  |
| cell line (*Homo sapiens*) | HEK293 FlpIn T-REx HASt-LTV1 | (Wyler et al., 2011) DOI: [10.1261/rna.2325911](https://doi.org/10.1261/rna.2325911) |  |  |
| cell line (*Homo sapiens*) | HEK293 FlpIn T-REx HASt-GFP | (Wyler et al., 2011) DOI: [10.1261/rna.2325911](https://doi.org/10.1261/rna.2325911) |  |  |
| cell line (*Homo sapiens*) | HEK293 FlpIn T-REx RIOK1-StHA | (Widmann et al., 2012) DOI: [10.1091/mbc.E11-07-0639](https://doi.org/10.1091/mbc.E11-07-0639) |  |  |
| cell line (*Homo sapiens*) | HEK293 FlpIn T-REx RIOK1(D324A)-StHA | (Widmann et al., 2012) DOI: [10.1091/mbc.E11-07-0639](https://doi.org/10.1091/mbc.E11-07-0639) |  |  |
| cell line (*Homo sapiens*) | HEK293 FlpIn T-REx RPS2-StHA | (Larburu et al., 2016) DOI: 10.1093/nar/gkw714 |  |  |
| cell line (*Homo sapiens*) | HEK293 FlpIn T-REx USP16-StHA | This paper |  | See Materials and Methods, *Cell lines, antibodies, and reagents* |
| cell line (*Homo sapiens*) | HEK293 FlpIn T-REx USP16(C205S)-StHA | This paper |  | See Materials and Methods, *Cell lines, antibodies, and reagents* |
| cell line (*Homo sapiens*) | HEK293 FlpIn T-REx USP16(ZnF)-StHA | This paper |  | ZnF: aa 1-192  See Materials and Methods, *Cell lines, antibodies, and reagent* |
| cell line (*Homo sapiens*) | HEK293 FlpIn T-REx USP16(USP)-StHA | This paper |  | USP: aa 193-822  See Materials and Methods, *Cell lines, antibodies, and reagents* |
| cell line (*Homo sapiens*) | HEK293 FlpIn T-REx USP16(ΔH)-StHA | This paper |  | ΔH: aa 1-822Δ436-460  See Materials and Methods, *Cell lines, antibodies, and reagents* |
| cell line (*Homo sapiens*) | HEK293 FlpIn T-REx USP16(USPΔH)-StHA | This paper |  | USPΔH: aa 193-822Δ436-460  See Materials and Methods, *Cell lines, antibodies, and reagents* |
| cell line (*Homo sapiens*) | HeLa FlpIn T-REx RPS27a-StHA | This paper |  | See Materials and Methods, *Cell lines, antibodies, and reagents* |
| cell line (*Homo sapiens*) | HeLa FlpIn T-REx RPS27a(7R)-StHA | This paper |  | 7R: K89/90/96/99/107/113/152R  See Materials and Methods, *Cell lines, antibodies, and reagents* |
| cell line (*Homo sapiens*) | HeLa FlpIn T-REx RPS27a(6R)-StHA | This paper |  | 6R: K89/90/96/99/107/152R  See Materials and Methods, *Cell lines, antibodies, and reagents* |
| cell line (*Homo sapiens*) | HeLa FlpIn T-REx RPS27a(K113R)-StHA | This paper |  | See Materials and Methods, *Cell lines, antibodies, and reagents* |
| cell line (*Homo sapiens*) | HeLa Kyoto | other | RRID:CVCL_1922 | Obtained from D. Gerlich (IMBA, Vienna). |
| cell line (*Homo sapiens*) | HeLa USP16 KO #1 | This paper |  | See Materials and Methods, *Generation of knockout cell lines* |
| cell line (*Homo sapiens*) | HeLa USP16 KO #2 | This paper |  | See Materials and Methods, *Generation of knockout cell lines* |
| cell line (*Homo sapiens*) | HEK293 FlpIn T-REx | Invitrogen | RRID:CVCL_U427 |  |
| cell line (*Homo sapiens*) | HEK293 FlpIn T-REx USP16 KO #1 | This paper |  | See Materials and Methods, *Generation of knockout cell lines* |
| cell line (*Homo sapiens*) | HEK293 FlpIn T-REx USP16 KO #2 | This paper |  | See Materials and Methods, *Generation of knockout cell lines* |
| transfected construct (human) | si-USP16-2 | Qiagen/Microsynth |  | 5’-AAUGGCUGAAAUAACGAUAAA-3’ |
| transfected construct (human) | si-USP16-3 | Microsynth; (Joo et al., 2007) DOI: [10.1038/nature06256](https://doi.org/10.1038/nature06256) |  | 5’-CCUCCUGUUCUUACUCUUCAUUUAA-3’ |
| transfected construct (human) | si-ZNF598-1 | Qiagen |  | 5’-CAGGACUACUACAGCGACUAU-3’ |
| transfected construct (human) | si-ZNF598-2 | Qiagen |  | 5’-ACAAAUGGTCCTGUAAGCCAA-3’ |
| transfected construct (human) | si-ZNF598-3 | Qiagen |  | 5’-UGGAAAGGUGUACGCAUUGUA-3’ |
| transfected construct (human) | si-ZNF598-4 | Qiagen |  | 5’-CACAGAUGUGUUGUGUAAACA-3’ |
| antibody | Anti-ATF4 (rabbit monoclonal) | Cell Signaling Technology | Cat# 11815  RRID:AB_2616025 | WB(1:1000) |
| antibody | Anti-β-actin (mouse monoclonal) | Sigma Aldrich | Cat# A1978  RRID:AB_476692 | WB(1:40’000) |
| antibody | Anti-cMYC (mouse monoclonal) | Santa Cruz Biotechnologies | Cat# sc-40  RRID:AB_627268 | WB(1:500) |
| antibody | Anti-DIM2 (rabbit polyclonal) | (Zemp et al., 2009) DOI: [10.1083/jcb.200904048](https://doi.org/10.1083/jcb.200904048) |  | IF(1:2000) |
| antibody | Anti-ENP1 (rabbit polyclonal) | (Zemp et al., 2009) DOI: [10.1083/jcb.200904048](https://doi.org/10.1083/jcb.200904048) |  | IF(1:15’000) |
| antibody | Anti-HA (mouse monoclonal) | Covance | Cat# MMS-101P  RRID:AB_2314672 | IF(1:3000)  WB(1:3000) |
| antibody | Anti-LTV1 (rabbit polyclonal) | (Zemp et al., 2009) DOI: [10.1083/jcb.200904048](https://doi.org/10.1083/jcb.200904048) |  | WB(1:2000) |
| antibody | Anti-NOB1 (rabbit polyclonal) | (Zemp et al., 2009) DOI: [10.1083/jcb.200904048](https://doi.org/10.1083/jcb.200904048) |  | IF(1:5000)  WB(1:2000) |
| antibody | Anti-NOC4L (rabbit polyclonal) | (Wyler et al., 2011) DOI: [10.1261/rna.2325911](https://doi.org/10.1261/rna.2325911) |  | WB(1:5000) |
| antibody | Anti-RIOK1 (rabbit polyclonal) | (Widmann et al., 2012) DOI: [10.1091/mbc.E11-07-0639](https://doi.org/10.1091/mbc.E11-07-0639) |  | IF(1:8000) |
| antibody | Anti-RIOK2 (rabbit polyclonal) | (Zemp et al., 2009) DOI: [10.1083/jcb.200904048](https://doi.org/10.1083/jcb.200904048) |  | IF(1:5000) |
| antibody | Anti-RLP24 (rabbit polyclonal) | (Wild et al., 2010) DOI: [10.1371/journal.pbio.1000522](https://doi.org/10.1371/journal.pbio.1000522) |  | WB(1:2500) |
| antibody | Anti-RPL5 (rabbit polyclonal) | Abcam | Cat# ab86863  RRID:AB_10671811 | WB(1:2000) |
| antibody | Anti-RPL23a (rabbit polyclonal) | (Wyler et al., 2011) DOI: [10.1261/rna.2325911](https://doi.org/10.1261/rna.2325911) |  | WB(1:200) |
| antibody | Anti-RPS2 (rabbit polyclonal) | This paper |  | WB(1:200)  See Materials and Methods, *Cell lines, antibodies, and reagents* |
| antibody | Anti-RPS3 (rabbit polyclonal) | (Zemp et al., 2009) DOI: [10.1083/jcb.200904048](https://doi.org/10.1083/jcb.200904048) |  | WB(1:1000) |
| antibody | Anti-RPS3a (rabbit polyclonal) | (Wyler et al., 2011) DOI: [10.1261/rna.2325911](https://doi.org/10.1261/rna.2325911) |  | WB(1:2000) |
| antibody | Anti-RPS10 (rabbit monoclonal) | Abcam | Cat# ab151550  RRID:AB_2714147 | WB(1:1000) |
| antibody | Anti-RPS20 (rabbit monoclonal) | Abcam | Cat# ab133776  RRID:AB_2714148 | WB(1:1000) |
| antibody | Anti-RPS27a (rabbit polyclonal) | This paper |  | WB(1:200)  See Materials and Methods, *Cell lines, antibodies, and reagents* |
| antibody | Anti-RRP12 (rabbit polyclonal) | (Wyler et al., 2011) DOI: [10.1261/rna.2325911](https://doi.org/10.1261/rna.2325911) |  | IF(1:2000) |
| antibody | Anti-TSR1 (rabbit polyclonal) | (Zemp et al., 2014) DOI: [10.1242/jcs.138719](https://doi.org/10.1242/jcs.138719) |  | WB(1:10’000) |
| antibody | Anti-ubiquitin (mouse monoclonal) | Santa Cruz Biotechnologies | Cat# sc-8017  RRID:AB_2762364 | WB(1:500) |
| antibody | Anti-USP16 (rabbit polyclonal) | Bethyl Laboratories | Cat# A301-615A  RRID:AB_1211387 | WB(1:500) |
| antibody | Anti-ZNF598 (rabbit polyclonal) | Abcam | Cat# ab80458  RRID:AB_2221273 | WB(1:1000) |
| recombinant DNA reagent | pcDNA5/FRT/TO/USP16-StHA | This paper |  | See Materials and Methods, *Molecular cloning* |
| recombinant DNA reagent | pcDNA5/FRT/TO/USP16(C205S)-StHA | This paper |  | See Materials and Methods, *Molecular cloning* |
| recombinant DNA reagent | pcDNA5/FRT/TO/USP16(ZnF)-StHA | This paper |  | ZnF: aa 1-192  See Materials and Methods, *Molecular cloning* |
| recombinant DNA reagent | pcDNA5/FRT/TO/USP16(USP)-StHA | This paper |  | USP: aa 193-822  See Materials and Methods, *Molecular cloning* |
| recombinant DNA reagent | pcDNA5/FRT/TO/USP16(ΔH)-StHA | This paper |  | ΔH: aa 1-822Δ436-460  See Materials and Methods, *Molecular cloning* |
| recombinant DNA reagent | pcDNA5/FRT/TO/USP16(USPΔH)-StHA | This paper |  | USPΔH: aa 193-822Δ436-460  See Materials and Methods, *Molecular cloning* |
| recombinant DNA reagent | pcDNA5/FRT/TO/RPS27a-StHA | This paper |  | See Materials and Methods, *Molecular cloning* |
| recombinant DNA reagent | pcDNA5/FRT/TO/RPS27a(7R)-StHA | This paper |  | 7R: K89/90/96/99/107/113/152R  See Materials and Methods, *Molecular cloning* |
| recombinant DNA reagent | pcDNA5/FRT/TO/RPS27a(6R)-StHA | This paper |  | 6R: K89/90/96/99/107/152R  See Materials and Methods, *Molecular cloning* |
| recombinant DNA reagent | pcDNA5/FRT/TO/RPS27a(K113R)-StHA | This paper |  | See Materials and Methods, *Molecular cloning* |
| recombinant DNA reagent | pC2P | (Welte et al., 2019) DOI: [10.1101/gad.328492.119](https://doi.org/10.1101/gad.328492.119) |  |  |
| recombinant DNA reagent | pC2P_USP16_gRNA_1 | This paper |  | Protospacer: 5’-TATTGTCAGTCTTACAGTCT-3’  See Materials and Methods, *Generation of knockout cell lines* |
| recombinant DNA reagent | pC2P_USP16_gRNA_2 | This paper |  | Protospacer: 5’-TATTGTCAGTCTTACAGTCT-3’  See Materials and Methods, *Molecular cloning* |
| sequence-based reagent | RPS27a(7R) | Thermo Fischer Scientific |  | 7R: K89/90/96/99/107/113/152R |
| sequence-based reagent | 5’ ITS1 probe | Microsynth; (Roquette et al., 2005) DOI: [10.1038/sj.emboj.7600752](https://doi.org/10.1038/sj.emboj.7600752) |  | Northern blot probe, 5′-CCTCGCCCTCCGGGCTCCGTTAATGATC -3′ |
| sequence-based reagent | si-control | Qiagen | Cat# 1027281 | Allstars siRNA |
| chemical compound, drug | cycloheximide, CHX | Sigma Aldrich | Cat# C7698 |  |
| chemical compound, drug | DTT | Applichem | Cat# A1101 |  |
| chemical compound, drug | G418 | Thermo Fischer Scientific | Cat# 11811 |  |
| chemical compound, drug | Leptomycin B, LMB | LC Laboratories | Cat# L-6100 |  |
| chemical compound, drug | MG132 | Sigma Aldrich | Cat# C2211 |  |
| chemical compound, drug | N-Ethylmaleimide, NEM | Sigma Aldrich | Cat# E3876 |  |
| chemical compound, drug | silvestrol | MedChemExpress | Cat# HY-13251 |  |
